# The problematic use of Information and Communication Technologies (ICT) in adolescents by the cross sectional JOITIC study

**DOI:** 10.1186/s12887-016-0674-y

**Published:** 2016-08-22

**Authors:** Raquel Muñoz-Miralles, Raquel Ortega-González, M. Rosa López-Morón, Carme Batalla-Martínez, Josep María Manresa, Núria Montellà-Jordana, Andrés Chamarro, Xavier Carbonell, Pere Torán-Monserrat

**Affiliations:** 1 Unitat de Suport a la Recerca Metropolitana Nord, Institut de Investigació en Atenció Primària (IDIAP) Jordi Gol, Sabadell, Barcelona Spain; 2 Departament d’Infermeria, Universitat Autònoma de Barcelona, Bellaterra, Barcelona Spain; 3 Àrea Bàsica de Salut Manresa 2, Institut Català de la Salut, Manresa, Barcelona Spain; 4 Centre d’Atenció Primària Santa Perpètua de Mogoda, Institut Català de la Salut, Santa Perpètua de Mogoda, Barcelona Spain; 5 Centre d’Atenció Primària Castellar, Institut Català de la Salut, Castellar del Vallès, Barcelona Spain; 6 Centre d’Atenció Primària Sant Quirze, Institut Català de la Salut, Sant Quirze del Vallès, Barcelona Spain; 7 Departament Psicologia Bàsica, Evolutiva i de l’Educació, Universitat Autònoma de Barcelona, Bellaterra, Barcelona Spain; 8 Facultat de Psicologia, Ciències de l’Educació i de l’Esport (FPCEE) Blanquerna, Universitat Ramon Llull, Barcelona, Spain

**Keywords:** Internet, Addictive behaviour, Mobile phone, Video games, Adolescent

## Abstract

**Background:**

The emerging field of Information and Communications Technology (ICT) has brought about new interaction styles. Its excessive use may lead to addictive behaviours.

The objective is to determine the prevalence of the problematic use of ICT such as Internet, mobile phones and video games, among adolescents enrolled in mandatory Secondary Education (ESO in Spanish) and to examine associated factors.

**Methods:**

Cross sectional, multi-centric descriptive study. Population: 5538 students enrolled in years one to four of ESO at 28 schools in the Vallès Occidental region (Barcelona, Spain). Data collection: self-administered socio-demographic and ICT access questionnaire, and validated questionnaires on experiences related to the use of the Internet, mobile phones and video games (CERI, CERM, CERV).

**Results:**

Questionnaires were collected from 5,538 adolescents between the ages of 12 and 20 (77.3 % of the total response), 48.6 % were females. Problematic use of the Internet was observed in 13.6 % of the surveyed individuals; problematic use of mobile phones in 2.4 % and problematic use in video games in 6.2 %.

Problematic Internet use was associated with female students, tobacco consumption, a background of binge drinking, the use of cannabis or other drugs, poor academic performance, poor family relationships and an intensive use of the computer.

Factors associated with the problematic use of mobile phones were the consumption of other drugs and an intensive use of these devices.

Frequent problems with video game use have been associated with male students, the consumption of other drugs, poor academic performance, poor family relationships and an intensive use of these games.

**Conclusions:**

This study offers information on the prevalence of addictive behaviours of the Internet, mobile phones and video game use.

The problematic use of these ICT devices has been related to the consumption of drugs, poor academic performance and poor family relationships.

This intensive use may constitute a risk marker for ICT addiction.

## Background

The expansion of the Information and Communication Technologies (ICT) in our society has resulted in numerous positive elements, including new means of communication, working, learning and entertainment, across space and time. Internet browsing, the use of social networks, video games and mobile phones have produced a radical lifestyle change, particularly amongst the youngest, also known as digital natives [1], who use these devices heavily. It has also led to problems associated with an inappropriate or excessive use, including work and school absenteeism, academic failure, deterioration of family or friendship relationships and even health problems [2–4], particularly among adolescents. It seems that the use of these technologies normalizes with age toward a more academic and less playful use, and with fewer negative consequences.

Information and Communication Technologies addiction has been highly argued over recent years, and the limits of appropriate use are still unclear. Various studies have aimed to quantify the magnitude of the inappropriate use of these technologies, with different results: 5 % for problems with Internet use [5, 6] 15,3 % [7], 9,4 % [8] or 34,7 % [9]; for problematic gaming between 2,7 % [10] and 9,3 % [11], 20 % for dependence with mobile phone [12]. Variability in the methods makes studies difficult to compare, as well the evolution of the definition of the disorder itself.

Among behavioural addictions, after the initial concern about Internet Addiction [13], technological addictions [14] have been an important focus of study. This field has also received increased attention after the DSM-5 considered Internet Gaming Disorder (IGD) in section III, as a disorder that requires further study [15] and some consensus seems to be gathered about the diagnosis criteria [16] although it is not exempt from some criticism [17, 18]. The following essential diagnostic elements may also be present in the abuse of the new technologies, particularly in the case of the Internet: psychological dependence, modification of mood, tolerance and abstinence, and adverse effects such as unjustified absenteeism or academic failure. Some studies have noted that adolescents who are addicted to the Internet, as in the case of drug addictions, present problems of aggression, anxiety, phobia, depression, sleep disorders and, in some cases, suffer from loneliness and social isolation [2, 3, 19, 20].

With mobile phones, these symptoms may also appear, although they tend to be less serious [3, 21, 22]. Similar symptoms also have been found with video games, particularly on-line games [10, 23], which may substitute human contact with virtual relationships. Clearly there are many similarities between drug addiction and some manifestations of ICT use, which is why they both elicit the frequent use of the term “addiction” but many literature on this topic use a term other than “addiction” for high-engagement with certain behaviours that do not fulfil all the criteria of classical addiction, but exhibit similar features. With this in mind, alternative terms for “addiction” such as “problematic use” have been proposed [24–27].

The objective of this study is to determine the prevalence of the problematic use of ICT in adolescent students, and to describe its association with the consumption of toxic substances, academic performance, family relationships and the intensity of ICT use.

## Methods

**Fig. 1:**
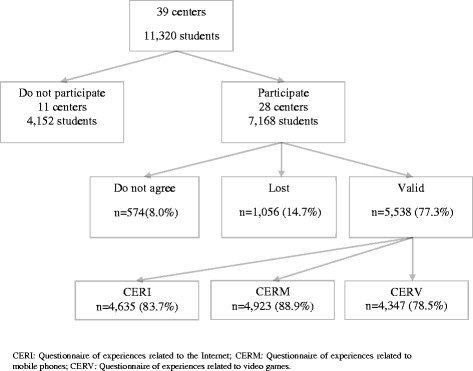
Flowchart of participating subjects

This is a descriptive, cross sectional and multi-centric study. The JOves I Tecnologies de la Informació i la Comunicació (JOITIC) study protocol was approved by the Clinical Research Ethics Committee of IDIAP Jordi Gol. The study population consisted of all of the students at the mandatory Secondary Education (ESO) enrolled in 2010–11 year. Participating schools were centres in which the “*Programa Salut i Escola*” (“Health and School Program” or PSiE, for its initials in Catalan) of the Catalonia government was being carried out. Of 11,320 students enrolled in the 39 centres of the metropolitan Barcelona region, 7,168 students between the ages of 12 and 20 were eligible from the 28 centres that agreed to participate [28] (Fig. 1). The liaison nurse from the PSiE provided the materials (informed consent forms and questionnaires) to the responsible parties of the centres. Students responded to anonymous questionnaires that were self-administered, regarding socio-demographic information and specific questionnaires on the ICT, during school hours and in the presence of their tutor. Tutors were supposed to support the activity but no intervention had to be done, neither any access to the answers or data.

The socio-demographic questionnaire [28] collected information regarding the following variables: age, gender, school year, type of centre (public-charter), participation in after-school activities, consumption of toxic substances (tobacco, alcohol, cannabis and other drugs), family relationships (referred by the student: «very bad» to «very good»), poor academic performance (three or more subjects failed during the previous school year), parental control of the type of ICT (control of use: yes or not) and intensive use consisting of 3 or more hours daily of computer use, over 5 h of video games per week and 10 or more SMS messages daily [29].

Patterns of use were identified via questionnaires that were specifically validated in accordance with technology: CERI (Questionnaire of experiences related to Internet use), CERM (Questionnaire of experiences related to mobile phones) [30] (Questionnaire of experiences related to video games) [31]. Questionnaires CERI and CERM contain 10 Likert items and 17 for CERV, with four possible answers scored from 1 to 4 (1: never/almost never, 2: occasionally, 3 sometimes, 4: almost always). The score result is the sum of responses for all items.

The reliability analysis of three questionnaires obtained Cronbach’s alpha values of 0.77 for CERI, 0.80 for CERM and 0.91 for CERV.

*”Problematic use”* was defined depending upon whether the score from the questionnaire was equal to or above 26 for the CERI, 24 for the CERM or 39 for the CERV and use with *“occasional problems”* was based upon a score between 18 and 25 for the CERI, 16–23 for the CERM or 26–38 for the CERV [30, 31].

### Statistical analysis

The categorical variables are described with absolute and relative frequencies. The quantitative ones are described by their mean and standard deviations.

In the contrasts for comparison of proportions, the Chi-square distribution or linear trend analysis was used.

Multivariable logistic regression was used for each of the examined technologies in order to explore what factors are related with their problematic use (dependent variable). Subsequently, new analyses were repeated to relate low academic performance (dependent variable) with the use of the ICT and other risk factors. All variables having a significance of *p* < 0.125 were considered to be candidates for evaluation in the creation of a final model for each technology, in which, after a manual process, only those having a significant OR or that modified the beta coefficients by more than 10 % were maintained.

Data analysis was carried out using the SPSS version 18.0 statistical package.

Given the large volume of participants, any small difference may be significant. Therefore, although the significance level used in all of the contrasts was *p* ≤ 0.001, the size of the observed associations has been considered to be relevant when the differences between groups were over 5 %.

## Results

Five hundred seventy four (8.0 %) parents and/or students did not agree to participate and 1,056 (14.7 %) answers got lost (students did not attend to the chosen class hour to administrate the questionnaire or did not answer it). 5,538 valid answers were collected (77.3 % responders of the initially included) from students between the ages of 12 and 20, 48.6 % of whom were females. The percentage of no responses in each of the socio-demographic questionnaires was less than 1%, except in academic performance (3.13 %). The number of questionnaires that were correctly completed differed based on questionnaire type (Fig. 1).

Based upon the cut off points established for the questionnaires, problematic Internet use was observed in 13.6 % of the students; problematic mobile phone use was seen in 2.4 %; and problematic video game use was found in 6.2 % (Table 1).

**Table 1:** Pattern of use of ICT

	No problems	Occasional problems	Problematic use
CERI	1917 (41.4 %)	2084 (45.0 %)	632 (13.6 %)
CERM	3977 (80.9 %)	822 (16.7 %)	119 (2.4 %)
CERV	2908 (66.9 %)	1167 (26.9 %)	269 (6.2 %)

*ICT* information and communication technologies, *CERI* questionnaire of experiences related to the internet, *CERM* questionnaire of experiences related to mobile phones, *CERV* questionnaire of experiences related to video games

In the analysis by technologies, problematic Internet use is found to be more frequent in females (17.0 %) as compared to males (10.6 %), with increases from the 1st to 3rd years of ESO, and decreases in the 4th year (Table 2). Tobacco use (27.1 vs 11.4 %), a history of binge drinking (23.4 vs 11.0 %), the use of cannabis (23.6 vs 11.9 %) or other drugs (31.3 vs 13.2 %) was also related to higher rates of addiction, as were poor academic performance (18.6 vs 12.3 %), poor family relationships (28.8 vs 11.7 %) and intensive computer use (>3 h/day) (35.8 vs 7.5 %).

**Table 2:** Bivariate analysis of individuals with problematic internet use and related factors

	CERI (*n* = 4635)			
	No problems	Occasional problems	Problematic use	*p*
Gender				<0.001
Females	826 (37.8 %)	988 (45.2 %)	371 (17.0 %)	
Males	1075 (44.6 %)	1078 (44.8 %)	255 (10.6 %)	
Type of center				<0.001
Public	1278 (39.7 %)	1461 (45.4 %)	478 (14.9 %)	
Charter	639 (45.1 %)	623 (43.9 %)	156 (11.0 %)	
Year				<0.001
1st	653 (49.7 %)	508 (38.7 %)	152 (11.6 %)	
2nd	467 (42.5 %)	484 (44.1 %)	147 (13.4 %)	
3rd	392 (32.9 %)	598 (50.2 %)	202 (16.9 %)	
4th	405 (39.3 %)	493 (47.8 %)	133 (12.9 %)	
After-school activities				<0.001
Yes	1493 (42.8 %)	1557 (44.6 %)	439 (12.6 %)	
No	416 (36.8 %)	523 (46.2 %)	192 (17.0 %)	
Poor academic performance				<0.001
Yes	293 (33.1 %)	428 (48.3 %)	165 (18.6 %)	
No	1574 (43.6 %)	1596 (44.2 %)	444 (12.3 %)	
Family relationship				<0.001
Good/very good	1791 (43.8 %)	1815 (44.4 %)	480 (11.7 %)	
Poor/indifferent	112 (22.2 %)	247 (49.0 %)	145 (28.8 %)	
Cigarettes				<0.001
Yes	176 (26.9 %)	300 (45.8 %)	177 (27.1 %)	
No	1741 (43.7 %)	1784 (44.8 %)	455 (11.4 %)	
Binge drinking at least once				<0.001
Yes	257 (26.1 %)	498 (50.6 %)	230 (23.4 %)	
No	1651 (45.6 %)	1571 (43.4 %)	398 (11.0 %)	
Cannabis				<0.001
Yes	175 (27.0 %)	320 (49.4 %)	153 (23.6 %)	
No	1728 (43.8 %)	1747 (44.3 %)	470 (11.9 %)	
Other drugs				<0.001
Yes	28 (25.0 %)	49 (43.8 %)	35 (31.3 %)	
No	1876 (41.8 %)	2018 (45.0 %)	590 (13.2 %)	
Intensive computer use				<0.001
≤ 3 h/day	1741 (48.7 %)	1567 (43.8 %)	267 (7.5 %)	
> 3 h/day	156 (15.3 %)	499 (49.0 %)	366 (35.8 %)	
Adult control				<0.001
Yes	1075 (44.7 %)	1049 (43.7 %)	280 (11.6 %)	
No	809 (37.8 %)	988 (46.2 %)	343 (16.0 %)	
Email				0.710
Yes	1274 (40.7 %)	1433 (45.7 %)	426 (13.6 %)	
No	581 (41.2 %)	628 (44.5 %)	201 (14.3 %)	
Chat				<0.001
Yes	754 (31.7 %)	1175 (49.4 %)	449 (18.9 %)	
No	1101 (50.9 %)	886 (40.9 %)	178 (8.2 %)	
Online games				0.384
Yes	618 (39.6 %)	729 (46.8 %)	212 (13.6 %)	
No	1237 (41.5 %	1332 (44.6 %)	415 (13.9 %)	
Social networks				<0.001
Yes	1465 (37.2 %)	1882 (47.7 %)	595 (15.1 %)	
No	390 (64.9 %)	179 (29.8 %)	32 (5.3 %)	
Scholastic information				<0.001
Yes	1054 (46.6 %)	968 (42.8 %)	239 (10.6 %)	
No	801 (35.1 %)	1093 (47.9 %)	388 (17.0 %)	
Purchases				<0.001
Yes	146 (33.2 %)	210 (47.7 %)	84 (19.1 %)	
No	1709 (41.7 %)	1851 (45.1 %)	543 (13.2 %)	

*CERI* questionnaire of experiences related to the internet

Increased problematic use was also found in those involved in Chats (18.9 vs 8.2 %), social networks (15.1 vs 5.3 %), non-academic use (17.0 vs 10.6 %) and those making purchases (19.1 vs 13.2 %).

A healthier use was found amongst those students who participated in after-school activities (42.8 vs 36.8 %) and those that made reference to adult control (44.7 vs 37.8 %). There was no relevant association observed with the remaining variables.

The problematic use of mobile phones was associated with drug use (14.3 vs 2.2 %) and the intensive use of this device (25.5 vs 1.9 %) (Table 3). Occasional problems were associated with the female gender (21.0 vs 12.4 %), the use of tobacco (30.2 vs 14.5 %), alcohol (26.8 vs 14.1 %), cannabis (26.6 vs 15.3 %), poor academic performance (25.5 vs 14.3 %), poor family relationships (26.3 vs 15.5 %), intensive mobile phone use (>10 SMS/day) (48.0 vs 16.2 %), the use of Chats (34.5 vs 15.3 %), games (25.9 vs 15.6 %) and the sending SMS (21.6 vs 10.7 %). No relevant association was observed with the drug use and phone calls.

**Table 3:** Bivariate analysis of the individuals with problematic use of mobile phones and related factors

	CERM (*n* = 4923)			
	No problems	Occasional problems	Problematic use	*p*
Gender				<0.001
Females	1820 (76.4 %)	501 (21.0 %)	62 (2.6 %)	
Males	2126 (85.3 %)	309 (12.4 %)	54 (2.2 %)	
Type of center				<0.001
Public	2711 (79.7 %)	592 (17.4 %)	99 (2.9 %)	
Charter	1266 (83.5 %)	230 (15.2 %)	20 (1.3 %)	
Year				0.001
1st	1173 (82.4 %)	207 (14.5 %)	43 (3.0 %)	
2nd	972 (83.6 %)	170 (14.6 %)	20 (1.7 %)	
3rd	974 (78.2 %)	241 (19.3 %)	31 (2.5 %)	
4th	857 (78.9 %)	204 (18.8 %)	25 (2.3 %)	
After-school activities				0.003
Yes	3032 (81.9 %)	580 (15.7 %)	91 (2.5 %)	
No	932 (77.8 %)	239 (19.9 %)	27 (2.3 %)	
Poor academic performance				<0.001
Yes	664 (70.9 %)	239 (25.5 %)	33 (3.5 %)	
No	3214 (83.6 %)	551 (14.3 %)	79 (2.1 %)	
Family relationship				<0.001
Good/very good	3578 (82.5 %)	674 (15.5 %)	83 (1.9 %)	
Poor/indifferent	360 (67.5 %)	140 (26.3 %)	33 (6.2 %)	
Cigarettes				<0.001
Yes	439 (63.8 %)	208 (30.2 %)	41 (6.0 %)	
No	3538 (83.6 %)	614 (14.5 %)	78 (1.8 %)	
Binge drinking at least once				<0.001
Yes	703 (68.3 %)	276 (26.8 %)	50 (4.9 %)	
No	3246 (84.1 %)	545 (14.1 %)	69 (1.8 %)	
Cannabis				<0.001
Yes	456 (67.9 %)	179 (26.6 %)	39 (5.8 %)	
No	3486 (82.8 %)	642 (15.3 %)	82 (1.9 %)	
Other drugs				<0.001
Yes	64 (61.0 %)	26 (24.8 %)	15 (14.3 %)	
No	3884 (81.3 %)	790 (16.5 %)	103 (2.2 %)	
Intensive mobile phone use				<0.001
≤ 10 SMS/day	3916 (81.9 %)	773 (16.2 %)	93 (1.9 %)	
> 10 SMS/day	26 (26.5 %)	47 (48.0 %)	25 (25.5 %)	
Calls				0.030
Yes	3441 (79.6 %)	781 (18.1 %)	103 (2.4 %)	
No	71 (71.7 %)	22 (22.2 %)	6 (6.1 %)	
Chats				<0.001
Yes	390 (59.0 %)	228 (34.5 %)	43 (6.5 %)	
No	3122 (83.0 %)	575 (15.3 %)	66 (1.8 %)	
Games				<0.001
Yes	777 (71.0 %)	284 (25.9 %)	34 (3.1 %)	
No	2735 (82.2 %)	519 (15.6 %)	75 (2.3 %)	
SMS				<0.001
Yes	2301 (76.0 %)	654 (21.6 %)	74 (2.4 %)	
No	1211 (86.8 %)	149 (10.7 %)	35 (2.5 %)	

*CERM* questionnaire of experiences related to mobile phones

In the analysis of video games, problematic use were observed in regards to the male gender (10.6 vs 1.4 %), poor academic performance (10.4 vs 5.1 %), poor family relationships (13.8 vs 5.3 %), the consumption of other drugs (16.0 vs 5.9 %) and the intense use of video games (>5 h/week) (26.1 vs 3.2 %). No relevant association was observed with the remaining variables (Table 4).

**Table 4:** Bivariate analysis of individuals with problematic use of video games and related factors

	CERV (*n* = 4347)			
	No problems	Occasional problems	Problematic use	*p*
Gender				<0.001
Females	1857 (88.8 %)	206 (9.8 %)	29 (1.4 %)	
Males	1028 (46.5 %)	948 (42.9 %)	235 (10.6 %)	
Type of center				0.001
Public	1991 (66.6 %)	784 (26.2 %)	213 (7.1 %)	
Charter	917 (67.6 %)	383 (28.2 %)	56 (4.1 %)	
Year				0.001
1st	802 (64.3 %)	370 (29.6 %)	76 (6.1 %)	
2nd	689 (65.6 %)	300 (28.6 %)	61 (5.8 %)	
3rd	761 (67.2 %)	283 (25.0 %)	88 (7.8 %)	
4th	656 (71.9 %)	213 (23.3 %)	44 (4.8 %)	
After-school activities				0.017
Yes	2178 (66.0 %)	921 (27.9 %)	200 (6.1 %)	
No	719 (69.9 %)	241 (23.4 %)	69 (6.7 %)	
Poor academic performance				<0.001
Yes	483 (60.6 %)	231 (29.0 %)	83 (10.4 %)	
No	2350 (68.3 %)	914 (26.6 %)	176 (5.1 %)	
Family relationship				<0.001
Good/very good	2614 (67.9 %)	1031 (26.8 %)	204 (5.3 %)	
Poor/indifferent	265 (57.9 %)	130 (28.4 %)	63 (13.8 %)	
Cigarettes				<0.001
Yes	424 (73.2 %)	114 (19.7 %)	41 (7.1 %)	
No	2484 (66.0 %)	1053 (28.0 %)	228 (6.1 %)	
Binge drinking at least once				<0.001
Yes	617 (69.5 %)	198 (22.3 %)	73 (8.2 %)	
No	2276 (66.3 %)	963 (28.0 %)	195 (5.7 %)	
Cannabis				0.095
Yes	391 (67.0 %)	146 (25.0 %)	47 (8.0 %)	
No	2497 (66.9 %)	1016 (27.2 %)	220 (5.9 %)	
Other drugs				<0.001
Yes	51 (54.3 %)	28 (29.8 %)	15 (16.0 %)	
No	2840 (67.3 %)	1131 (26.8 %)	251 (5.9 %)	
Intensive video game use				<0.001
≤ 5 h/week	2750 (73.5 %)	873 (23.3 %)	119 (3.2 %)	
> 5 h/week	122 (22.0 %)	288 (51.9 %)	145 (26.1 %)	
Adult control of video game time				<0.001
Yes	1095 (58.5 %)	660 (35.3 %)	116 (6.2 %)	
No	1729 (72.9 %)	493 (20.8 %)	151 (6.4 %)	
Adult control of video game type				0.025
Yes	940 (65.1 %)	428 (29.5 %)	78 (5.4 %)	
No	1887 (67.1 %)	734 (26.1 %)	191 (6.8 %)	
Plays alone				<0.001
Yes	1121 (57.3 %)	672 (34.3 %)	165 (8.4 %)	
No	1621 (73.9 %)	470 (21.4 %)	103 (4.7 %)	

*CERV* Questionnaire of experiences related to video games

The presence of occasional or frequent problems in students in the first cycle (1st and 2nd year) as compared to the 2nd cycle (3rd and 4th year of ESO) increased for Internet use by 53.5 vs 64.1 % (*p* < 0.001) and for mobile phone use, by 17.0 vs 21.5 % (*p* < 0.001), but decreased for video game use from 35.1 vs 30.7 % (*p* < 0.001).

In the multivariate analysis, the problematic use of the Internet was associated with the female gender (*OR* = 1.49), tobacco consumption (*OR* = 1.55), binge drinking (*OR* = 1.35), poor family relationships (*OR* = 2.05) and intensive use (>3 h/day) (*OR* = 5.77) (Table 5). Problematic use of mobile phones is associated with tobacco consumption (*OR* = 2.16), with poor family relationships (*OR* = 2.33) and intensive use (sending >10 SMS messages/day) (*OR* = 12.39). As for video game use, males had a higher risk of problematic use (*OR* = 4.63), as did students with poor family relationships (*OR* = 2.82), those engaging in intensive use (>5 h/day) (*OR* = 6.90) and those who play alone (*OR* = 1.66).

**Table 5:** Exploratory models of multivariate logistic regression to associate potential risk factors with the presence of regular problems in the use of the Internet, mobile phones and video games

Internet	Coefficient	OR (CI 95 %)	*p*
Males	0.397	1.49 (1.26–1.79)	<0.001
Smoking	0.435	1.55 (1.20–1.99)	0.001
Binge drinking	0.303	1.35 (1.08–1.71)	0.010
Poor relationship with family	0.718	2.05 (1.61–2.62)	<0.001
Computer time (>3 h)	1.752	5.77 (4.8–6.96)	<0.001
Constant	−2.943		
Mobile phone	Coefficient	OR (CI 95 %)	*p*
Smoking	0.771	2.16 (1.41–3.33)	<0.001
Poor relationship with family	0.847	2.33 (1.49–3.66)	<0.001
SMS (>10)	2.516	12.39 (7.32–20.97)	<0.001
Constant	−4.225		
Video games	Coefficient	OR (CI 95 %)	*p*
Male	−1.533	0.22 (0.14–0.33)	<0.001
Poor relationship with family	1.036	2.82 (1.98–4.01)	<0.001
Time with video games (>5 h)	1.932	6.902 (5.21–9.14)	<0.001
Plays alone	0.508	1.66 (1.25–2.20)	0.001
Constant	−4.810		

OR (CI 95 %): Odds Ratio and 95 % Confidence Intervals

Upon creating new models of logistic regression using poor academic performance as the dependent variable, we find that female gender, good family relationships and participation in after-school activities are protective factors, while the consumption of toxic substances is a risk factor (Table 6).

**Table 6:** Exploratory models of multivariate logistic regression related with poor academic performance (dependent variable)

Internet	Coefficient	OR (CI 95 %)	*p*
Female	−0.682	0.51 – 0.43–0.60)	<0.001
Good relationship with family	−0.567	0.57 – 0.45–0.71)	<0.001
Binge drinking	0.398	1.49 1.20–1.85)	<0.001
Cannabis	0.485	1.62 (1.27–2.09)	<0.001
Smoking	0.820	2.27 (1.78–2.90)	<0.001
After-school activities			
1 day	−0.760	0.47 (0.33–0.66)	<0.001
2 days	−0.702	0.50 (0.40–0.62)	<0.001
3 days	−0.833	0.44 (0.36–0.53)	<0.001
Problematic use of Internet			
Occasional problems	0.219	1.25 (1.04–1.49)	0.016
Frequent problems	0.299	1.348 (1.053–1.727)	0.018
Constant	−0.554	0.58	<0.001
Mobile phones	Coefficient	OR (CI 95 %)	*p*
Female	−0.787	0.46 (0.39–0.54)	<0.001
Good relationship with family	−0.690	0.50 (0.40–0.63)	<0.001
Binge drinking	0.370	1.45 (1.17–1.79)	0.001
Cannabis	0.374	1.45 (1.13–1.87)	0.003
Smoking	0.843	2.32 (1.83–3.00)	<0.001
After-school activities			
1 day	−0.733	0.48 (0.34–0.67)	<0.001
2 days	−0.658	0.52 (0.42–0.65)	<0.001
3 days	−0.876	0.416 (0.34–0.50)	<0.001
Problematic use of mobile phone			
Occasional problems	0.611	1.843 (1.52–2.24)	<0.001
Frequent problems	0.273	1.314 (0.82–2.10)	0.254
Constant	−0.359	0.699	0.006
Video games	Coefficient	OR (CI 95 %)	*p*
Female	−0.704	0.49 (0.41–0.60)	<0.001
Good relationship with family	−0.645	0.53 (0.41–0.67)	<0.001
Binge drinking	0.392	1.48 (1.17–1.87)	0.001
Cannabis	0.406	1.50 (1.15–1.97)	0.003
Smoking	0.954	2.60 (2.00–3.37)	<0.001
After-school activities			
1 day	−0.725	0.48 (0.34–0.69)	<0.001
2 days	−0.678	0.51 (0.40–0.64)	<0.001
3 days	−0.906	0.40 (0.33–0.50)	<0.001
Problematic use of video games			
Occasional problems	0.042	1.04 (0.85–1.28)	0.692
Frequent problems	0.483	1.62 (1.18–2.23)	0.003
Constant	−0.413	0.66	0.009

Students with occasional or frequent problems with Internet use present the greatest risk for poor academic performance, although this exceeds our significance level (*p* > 0,001). For mobile phones, only those with occasional problems and for video games, only those having frequent problems posed this increased risk (Table 6).

## Discussion

We have obtained information about the prevalence of problematic use of mobile, Internet and video games on adolescents and examined risk factors. Selection of the participating study population and the high response percentage provide a realistic view of the degree of ICT problematic use in adolescents.

Internet addiction in adolescents is a topic of great social and familiar concern. In our study, 13.6 % of the surveyed individuals present problematic behaviour that is associated with this technology. This prevalence is similar to that which was reported by Yen in females [32], although in males it is much higher. In 2010, Carbonell et al. did not find differences and our study has revealed a greater frequency of problems in the females [33]. Most likely, this trend is related to the type of use which in a very short time, has evolved to the increased use of social networks, which tend to be used more frequently by females [34–36]. However, other studies have indicated that female adolescent or university-aged students are more aware of the risk, which should serve as a protective factor [29, 37].

The number of hours invested in Internet, videogames or mobile phone activities is not a definitive criterion in the diagnosis of technological addictions. In fact, researchers distinguish between high engagement and problematic use [38, 39] and suggest that some past studies may have overestimated the prevalence of addiction type problems of ICT users. Therefore the questionnaires like CERI and CERM are based on the negative consequences rather than in the time invested in ICT [30]. However, we have found a strong relationship between intensive use and problematic use as happens in other studies with video gamers [40] and Internet users [41] while the type of use disappears as an additional risk factor upon adjustments made via multivariate analysis. These results seem to indicate that for the youngest users, the number of hours of use is actually a risk factor. Poor family relationships appear as the second most important risk factor. Here, the role of the family as a regulator of use, may be fundamental for preventing Internet addiction [32, 42].

Drug use and impulsivity have been related with problematic Internet behaviour [43]. In our case, we have found associations with tobacco use and a history of binge drinking. As for mobile phones, an increased risk in problematic use has been found only in those that display intensive use of mobile phones or who consume other drugs. These results are consistent with findings from prior studies [6, 44]. Intensive use or the consumption of other drugs has also been associated with the problematic use of video games, as occurs with the male gender, poor academic performance and poor family relationships [45]. The multivariate analysis of logistic regression explores the role played by each of the variables in the problematic use of each ICT when combined with other variables [32, 42].

The risk of problematic use of mobile phones is similar to other studies [37, 46]. It is greatest in the public school students, as well as in those who use tobacco, have poor family relationships and that send more than 10 SMS messages per day [29]. While we are unaware of the association mechanism for type of school with problematic mobile phone behaviour, is may be related to socioeconomic status. Tobacco may constitute a group socialization marker. Once again, the main risk factor is intensity of use, measured as the number of SMS messages. Clearly, today SMS text messages would be substituted by WhatsApp messages. Our data suggest that, comparing to Internet and video games, there is a scarce evidence for considering mobile use as a problematic behaviour [22]. The adolescent not considered video games, which generate intense social alarm, as problematic as other ICT [37]. In our case, the prevalence rate of problematic use of video games found in the present study (6.2 %) indicates a highly comparable prevalence than those found in other European countries [10, 47, 48].

Those students whose parents controlled their game playing time had more occasional problems with this technology. We feel that this may be explained as a reaction to the intensification of playing time in children by parents who are more sensitized and active in the control of video game use. In the multivariate analysis, problematic use was associated with the profile of a male student, solitary player, dedicating many hours to the game, and having poor family relationships.

Based on the results obtained, data suggest that in all of the analysed ICT, intensive use is a good marker of addiction, regardless of the type of use that is engaged in. Similarly, poor family relationships appear to be an important risk factor for ICT problems.

As for academic failure, we feel that this may be a good indicator of the effect of the use of the new technologies in adolescents, although there are authors who have observed more failure in youth who do not use computers [49]. In our case, it has been associated with the presence of frequent problems with Internet and video game use and occasional problems with mobile phone use. In an earlier article, we reported the relationship between low academic performance and the intensive use of the ICT [28].

In the multivariate analysis, poor academic performance appears to be related with a combination of consumption of toxic substances and the moderate or frequent use of the Internet, mobile phones or video games. Being female, having good family relationships and participating in after-school activities appear as protective factors (Table 6).

These findings suggest that in adolescents, the problematic use of ICT is a risk factor for academic failure, in addition to others that may be inherent in this evolutionary stage such as starting to consume toxic substances. This damaging effect is possibly related to the interference and imbalance caused in the acquisition of study habits. Therefore, we agree with other authors that during the 1st cycle of ESO, it is necessary to undertake more future preventative actions in this area [5, 30].

### Limitations

Although the transversal design of the study does not permit the establishment of causality between the variables, we have generated hypotheses that should be examined in future longitudinal studies. In fact, despite certain variability in some results, there is a considerable agreement found with other studies. The use of different validated instruments to evaluate the problematic use of Internet, video games and mobile phones and the variety of cultural contexts prevents the comparison between studies.

Since this was a self-administered study, it is possible that under-declaration took place for those behaviours that are considered to be socially negative, as is the case with academic failure or drug consumption. It is possible that some losses correspond to individuals with an at-risk profile, although data collection was carried out during the academic day in order to minimize this possibility.

The fast evolution of the ICT has limited the study’s future validity, since current mobile phone devices already permit access to the Internet, interaction in the social networks and on-line games, which at the time of this study were at very early stages.

## Conclusions

Of the surveyed adolescents, 13.6 % presented addictive behaviour in regards to the Internet. However, the prevalence with respect to mobile phones and video games was quite lower.

The coexistence of problematic ICT use with the use of drugs, intensive use of technology, poor family relationships and poor academic performance was observed.

Intensive use was a good marker of problematic use of the ICT.

The role of the family may be fundamental in prevention efforts.

Interventions at an early age may be necessary in order to strengthen a healthy adolescent relationship with the ICT, primarily with the Internet.

## Abbreviations

CEIC: “*Comitè d’Ètica en Investigació Clínica*” (Clinical Research Ethics Committee)
CERI: “*Cuestionario de Experiencias Relacionadas con Internet*” (Questionnaire of experiences related to the Internet).
CERM: “*Cuestionario de Experiencias Relacionadas con el Móvil*” (Questionnaire of experiences related to mobile phones).
CERV: “*Cuestionario de Experiencias Relacionadas con los Videojuegos*” (Questionnaire of experiences related to video games).
ESO: “*Educació Secundària Obligatòria*” (Compulsory Secondary School).
IAT: Internet addiction test
ICT: Information and communication technologies
IDIAP Jordi Gol: “*Institut d’Investigació en Atenció Primària Jordi Gol*” (Primary Health Care Institute of Research)
IES: “*Institut d’Educació Secundària*” (Secondary High School)
JOITIC: “*JOves I Tecnologies de la Informació i la Comunicació*” (Youth and Information and Communication Technologies)
OR (CI95 %): Odds ratio and 95 % Confidence interval
OR: Odds ratio
PSiE: “*Programa Salut i Escola*” (Health and School program)
SMS: Short message service
SPSS: Statistical package for the social sciences
